# To construct the norm of hand hygiene behavior scale for medical staff in tertiary general hospitals in China

**DOI:** 10.3389/fmed.2025.1656547

**Published:** 2025-10-24

**Authors:** Juan Tao, Shuang-yan Liu, Si-ping Tang, Ye Yu, Sheng-yu Xiang

**Affiliations:** Nursing Department, Sir Run Run Shaw Hospital, Zhejiang University School of Medicine, Hangzhou, China

**Keywords:** medical staff, hand hygiene, norm, hand hygiene behavior scale, nursing—education

## Abstract

**Objective:**

This study aimed to construct a national norm for the Chinese version of the Hand Hygiene Behavior Questionnaire (HHBQ-C) among medical staff in tertiary general hospitals in China and to provide a basis for assessing the current status and influencing factors of hand hygiene practices among medical staff.

**Methods:**

Using a stratified random sampling method, a total of 3,000 medical staff from 12 provinces across three regions of China, eastern, central, and western, were selected as research subjects between April 2023 and June 2023. Each region included four provinces. The General Information Questionnaire and the HHBQ-C were used to evaluate the medical staff. Norms for means, percentiles, demarcation, and classification were established.

**Results:**

A total of 2,793 medical staff were included in this study. Gender and age were used to establish the mean norms of the HHBQ-C. Based on the results of the single-factor analysis, classification norms were established by selecting the characteristics that showed significant differences, namely age, occupation, work pressure, and workload. Percentile norms were established at 5% intervals. The scores on the HHBQ-C were divided into four grades: low, medium, high, and very high.

**Conclusion:**

The established national norms for the HHBQ-C in tertiary hospitals provide a solid basis for evaluating influencing factors and guiding targeted interventions to improve hand hygiene compliance and reduce HCAIs.

## Introduction

Health care-associated infections (HCAIs) are common clinical adverse events that threaten patient safety, cause serious complications, and raise mortality rates (1). According to the World Health Organization, Europe reports up to 2.6 million HCAI cases annually, leading to over 91,000 deaths, while the U. S. sees around 1.7 million cases and 99,000 deaths each year (2). The occurrence of HCAIs represents a significant contributor to additional healthcare costs in China, with studies indicating a resultant surge in per-hospitalization expenses of around 50% (3). This added financial burden profoundly impacts both patients and the sustainability of healthcare institutions.

Although studies confirm that poor hand hygiene compliance among medical staff significantly contributes to HCAIs (4, 5), global adherence to hand hygiene practices remains inadequate. A number of studies have shown that the hand hygiene compliance of medical staff is less than 70%, hand hygiene compliance among healthcare workers in China is consistent with this dataset (6, 7). Determinants of hand hygiene compliance among healthcare professionals differ significantly based on geographical, institutional, and demographic factors, therefore, it is crucial to comprehensively identify the factors influencing hand hygiene behaviors (HHB) among medical staff. The Chinese version of the HHB Questionnaire (HHBQ-C) (8), which was previously translated by our research group, can effectively identify the factors influencing HHB among medical staff.

However, a national standard for the HHBQ-C has not yet been established, creating challenges in screening and identifying these influencing factors. Consequently, our research group conducted a study to develop norms for the HHBQ-C among medical staff in tertiary general hospitals in China. The goal was to classify assessment grades for factors influencing HHB and to provide a reference for formulating targeted intervention strategies aimed at improving hand hygiene compliance among medical staff and thereby reducing the incidence of HCAIs.

## Objects and methods

### Subjects of study

A stratified random sampling method was used to collect samples from the three geographical regions of China: eastern (Liaoning, Jiangsu, Zhejiang, Shandong), central (Shanxi, Anhui, Henan, Hunan), and western (Sichuan, Guizhou, Shaanxi, Xinjiang) from April 2023 to June 2023. In each of the 12 provinces, two tertiary general hospitals were randomly selected. Medical staff working in the outpatient and emergency departments, internal medicine, surgery, ICU, and operating room were randomly chosen for investigation. Referring to the national norm sample size range (2,500–4,000) and considering the workload, the sample size was ultimately determined to be 3,000 (9–11). Inclusion criteria included doctors, nurses, medical technicians, and other medical personnel holding practicing qualification certificates. Exclusion criteria encompassed individuals who were on leave due to illness, maternity, or other reasons. The purpose of this study was fully explained, and informed consent was obtained from all participants before the survey.

## Methods

### Research tools

General information questionnaire: The general information questionnaire was developed by the research team based on previous literature (12, 13). It included items such as gender, age, education, occupation, years of work experience, department, professional title, whether the participant was a clinical teacher, and the influence of work-related pressure.

HHBQ-C: Lydon et al. (14) developed the Hand Hygiene Behavior Questionnaire (HHBQ) in 2019 based on the capability, opportunity, motivation-behavior (COM-B) model, aiming to comprehensively evaluate the factors influencing the HHB of medical staff. In 2022, the HHBQ was sinicized to form the HHBQ-C, which contains three dimensions: hand hygiene ability (5 items), hand hygiene opportunity (7 items), and hand hygiene motivation (5 items), for a total of 17 items. The items of the HHBQ-C were rated on a 5-point Likert scale, ranging from “strongly disagree” to “strongly agree,” with scores assigned from 1 to 5 points. The total score ranged from 17 to 85 points, with higher scores indicating fewer barriers and more promoting factors for HHB among medical staff (8). According to the formula: Percentage Score = (Original Score - Minimum Scale Score) / (Maximum Scale Score - Minimum Scale Score) × 100, the original scores were converted into percentage scores. The content validity index of each item ranged from 0.923 to 1.000, and the content validity index of the total scale was 0.995. The Cronbach’s α coefficient for each dimension ranged from 0.871 to 0.906, and the Cronbach’s α coefficient for the total scale was 0.918. The test–retest reliability of the scale was 0.739. These results demonstrate that the scale has good reliability and validity (8).

### Survey methods

This study adopted an online questionnaire survey method. The questionnaire was prepared and published through the Questionnaire Star platform. After providing informed consent, the respondents accessed the Questionnaire Star interface to complete the questionnaire, and the electronic data were stored in a database. Before the survey, the research team systematically studied the HHBQ-C and standardized the instructions for completing the form. Additionally, standardized instructions were included at the beginning of the questionnaire to ensure anonymity. The research team communicated thoroughly with the liaison staff of each hospital regarding the content and procedures of the questionnaire. Before the official release of the questionnaire, the research team issued uniform guidelines for completion. During the survey process, any questions raised by the participants were addressed and resolved consistently. Due to the large number of regions and hospitals involved in the survey, the questionnaire collection period was set at 3 months. To improve data quality, the system was configured so that each IP address could submit a response only once, and all items were required to be completed before submission. In the data analysis stage, the correlation items before and after the questionnaire were checked using logical consistency tests. Invalid questionnaires that contained obvious inconsistencies or exhibited clear patterns were excluded.

### Modeling methods

Based on the above survey results, this study established the mean, classification, percentile, and cut-off norms of the HHBQ-C for medical staff in China. The mean norm was expressed as ($\bar{x}$ ± s). The classification norm was developed based on the scale scores and the results of the general data analysis. Percentile norms were established by calculating percentiles. These three methods can identify the relative position of scores reflecting the influencing factors of hand hygiene among medical staff in the population, but cannot determine the specific score levels of these influencing factors (15). Given the large sample size of this study and the absence of a gold standard for evaluating the influencing factors of hand hygiene, the normal distribution method was selected (16) from among the commonly used approaches: the percentile method, the normal distribution method, and the receiver operating characteristic curve, for constructing the demarcation norm. Referring to previous studies (17) on model construction, different cut-off values within the range of ($\bar{x}$ ± 2.5 s) were calculated at intervals of 0.5 times the standard deviation, and the HHBQ-C assessment scores were divided into four levels: low, medium, high, and very high.

### Statistical methods

SPSS version 25.0 software was used for statistical analysis. Measurement data were expressed as the mean ± standard deviation, and count data were presented as frequencies and percentages. A *t* test or one-way analysis of variance was used to determine the classification norm, while Spearman rank correlation analysis was applied to determine the cut-off norm. A *p* value < 0.05 was considered statistically significant.

## Results

### General information of respondents

A total of 3,000 questionnaires were collected in this study; among them, 207 questionnaires that were illogical or showed obvious patterns were excluded. Consequently, 2,793 valid questionnaires were retained, resulting in an effective recovery rate of 93.1%. The average age of the respondents was (34.88 ± 7.69) years. Among them, 2,504 were female and 289 were male. Additional demographic information about the respondents is presented in Table 1.

**Table 1:** General information of the subjects (*n* = 2,793).

Items		Number of people	Percentage %
Gender	Female	2,504	89.65
	Male	289	10.35
Age	≤30	881	31.54
	31 ~	1,361	48.73
	>40	551	19.73
Highest degree	PhD	9	0.32
	Master’s	162	5.80
	Undergraduate	2,299	82.31
	College or below	323	11.56
Your occupation	Doctor	325	11.64
	Nurse	2,416	86.50
	Others	52	1.86
Years of service	≤5	757	27.10
	6 ~	624	22.34
	11 ~	377	13.50
	16 ~	408	14.61
	>20	712	15.09
Department	Internal medicine	724	25.92
	Surgery	924	33.08
	Intensive care	143	5.12
	Operating room	236	8.45
	Outpatient and emergency departments	207	7.41
	Other	559	20.01
Professional technical titles	Junior	1,067	38.20
	Intermediate	1,322	47.33
	Sub-high	301	10.78
	Direct height	48	1.72
	Other	55	1.97
Religious beliefs	No	2,704	96.81
	Islam	6	0.21
	Christianity	49	1.75
	Buddhism	16	0.57
	Others	18	0.64
Are you a clinical teacher?	Is	1,335	47.80
	No	1,458	52.20
A member of the hospital infection prevention and control team?	Is	576	20.62
	No	2,217	79.38
Do you think work stress affects your hand hygiene behavior?	A very big effect	552	19.76
	Minor impact	793	28.39
	Not much	652	23.34
	No impact	760	27.21
	Uncertain	36	1.29
Do you think the workload has an effect on your hand hygiene behavior?	A very big impact	599	21.45
	Minor impact	1,119	40.06
	Not much	563	20.16
	No impact	491	17.58
	Uncertain	21	0.75

### Mean norms of HHBQ-C among medical staff in tertiary general hospitals in China

The total HHBQ-C score of medical staff in tertiary general hospitals in China was (86.43 ± 15.27). The scores for the ability, opportunity, and motivation dimensions were (87.58 ± 18.05), (84.29 ± 16.55), and (88.27 ± 15.63), respectively. In addition, the medical staff were grouped by gender and age, and the mean HHBQ-C norms were established, as shown in Table 2.

**Table 2:** Mean norm of HHBQ-C (score, $\bar{x}$ ± s).

Gender	Age (years)	Overall score	Ability score	Opportunity score	Motivation score
Female	≤30	87.40 ± 14.70	87.66 ± 18.04	85.98 ± 15.67	89.12 ± 14.92
	31 ~	86.36 ± 16.24	87.87 ± 18.27	83.92 ± 17.62	88.26 ± 16.75
	Female > 40	86.47 ± 12.50	88.49 ± 16.37	83.48 ± 14.35	88.64 ± 12.53
	Total	86.70 ± 15.10	87.93 ± 17.83	84.47 ± 16.45	88.60 ± 15.43
Male	≤30	84.60 ± 17.15	83.73 ± 22.83	83.89 ± 17.90	86.46 ± 17.11
	31 ~	83.24 ± 14.73	84.62 ± 18.32	81.23 ± 15.27	84.68 ± 16.16
	Male > 35	84.09 ± 17.22	85.29 ± 17.09	82.69 ± 18.30	84.86 ± 17.57
	Total	84.05 ± 16.51	84.53 ± 19.64	82.74 ± 17.35	85.40 ± 16.99
Total	≤30	87.06 ± 15.03	87.19 ± 18.71	85.73 ± 15.96	88.80 ± 15.21
	31 ~	86.17 ± 16.17	87.68 ± 18.28	83.76 ± 17.49	88.05 ± 16.73
	>40 for women and >35 for men	86.05 ± 13.47	87.93 ± 16.53	83.34 ± 15.11	87.98 ± 13.61
	Total	86.43 ± 15.27	87.58 ± 18.05	84.29 ± 16.55	88.27 ± 15.63
Total amount	≤30	87.06 ± 15.03	87.19 ± 18.71	87.73 ± 15.96	88.80 ± 15.21
	31 ~	86.17 ± 16.08	87.69 ± 18.10	83.76 ± 17.45	88.03 ± 16.62
	>40	86.04 ± 13.47	87.90 ± 16.85	83.30 ± 15.02	88.00 ± 13.63
	Total	86.43 ± 15.27	87.58 ± 18.05	84.29 ± 16.55	88.27 ± 15.63

### HHBQ-C classification norm of medical staff in tertiary general hospitals in China

An independent sample’s *t* test and one-way analysis of variance showed that there were statistically significant differences in the HHBQ-C scores of medical staff across different occupations (*F* = 4.140, *p* = 0.016), levels of work stress (*F* = 16.801, *p <* 0.001) and workload (*F* = 14.0, *p* < 0.001), as shown in Table 3.

**Table 3:** Classification norm of HHBQ-C (score, $\bar{x}$ ± s).

Items		Overall score
Gender	Female	86.70 ± 15.10
	Male	84.05 ± 16.51
*t*		2.802
*P*		0.005
Age	≤30	87.06 ± 15.03
	31 ~	86.17 ± 16.08
	>40	86.04 ± 13.47
*F*		1.122
*P*		0.326
Highest degree	PhD	85.62 ± 14.63
	Master’s	84.86 ± 13.67
	Undergraduate	86.43 ± 15.60
	College or below	87.25 ± 13.55
*F*		0.889
*P*		0.446
Your occupation	Doctor ①	84.95 ± 15.58
	Nurse ②	86.72 ± 15.14
	Other ③	82.01 ± 18.02
*F*		4.140
*P*		0.016
Pairwise comparisons		②>③*
Years of service	≤5	86.60 ± 15.96
	6 ~	86.76 ± 15.01
	11 ~	86.21 ± 16.52
	16 ~	86.48 ± 15.54
	>20	85.82 ± 13.24
*F*		0.31
*P*		0.4874
Department	Internal medicine ①	86.52 ± 15.06
	Surgery ②	87.01 ± 15.51
	Intensive care ③	83.26 ± 18.54
	Operating room ④	86.81 ± 14.13
	Outpatient and emergency departments⑤	87.36 ± 12.94
	Other ⑥	85.65 ± 15.39
*F*		1.989
*P*		0.077
Professional technical titles	Junior ①	86.55 ± 16.06
	Intermediate 2	86.51 ± 15.02
	Sub-high ③	85.65 ± 13.43
	Direct height ④	84.74 ± 17.89
	Other ⑤	87.86 ± 12.39
*F*		0.490
*P*		0.743
Religious beliefs	No	86.41 ± 15.37
	Islam	84.31 ± 10.05
	Christianity	88.30 ± 11.76
	Buddhism	86.49 ± 13.54
	Others	84.48 ± 10.57
*F*		0.286
*P*		0.887
Are you a clinical teacher?	Is	86.08 ± 15.37
	No	86.75 ± 15.17
*t*		0.1155
*P*		0.248
A member of the hospital infection prevention and control team?	Is	87.12 ± 13.69
	No	86.25 ± 15.65
*t*		1.231
*P*		0.219
Do you think work stress affects your hand hygiene behavior?	A very big impact ①	86.68 ± 17.49
	Minor impact②	83.32 ± 16.02
	Not much ③	86.46 ± 12.73
	No effect ④	89.56 ± 13.47
	Uncertain ⑤	84.15 ± 24.26
*F*		16.801
*P*		*P* < 0.001
Pairwise comparisons		①>②***; ①>③**; ②<③***
		②<④***; ③<④***; ④>⑤*
Do you think the workload has an effect on your hand hygiene behavior?	A very big impact ①	84.88 ± 16.66
	Minor impact ②	85.03 ± 15.36
	Not much③	87.05 ± 13.67
	No effect ④	90.66 ± 14.13
	Uncertain ⑤	89.29 ± 16.50
*F*		14.01
*P*		*P* < 0.001
Pairwise comparisons		①<③*; ①<④***; ②<③*; ②<④***
		③<④***

*P < 0.05; **P < 0.01; ***P < 0.001.

### HHBQ-C percentile norm of medical staff in tertiary general hospitals in China

In this study, the total score of the scale was divided into intervals corresponding to 5% percentiles, and the percentile norms of the HHBQ-C from the 5th to the 95th percentile were established, as shown in Table 4.

**Table 4:** Percentile norms for HHBQ-C.

Percentiles	Female			Male		
	≤30	31 ~	>40	≤30	31 ~	>35
P5	68.82	66.18	69.12	62.28	67.43	66.32
P10	75.00	72.06	72.65	71.62	72.06	75.00
P15 ~ P25	75.00	75.00	75.00	75.00	75.00	75.00
P30	75.00	76.47	75.00	75.00	75.00	75.00
P35	79.41	80.88	79.41	75.66	75.00	75.00
P40	83.82	83.82	82.35	77.65	75.00	76.47
P45	88.53	88.24	86.76	80.88	77.28	77.94
P50	92.65	91.18	88.24	82.35	80.88	82.35
P55	95.59	94.12	91.18	89.26	85.15	88.24
P60	98.53	95.59	93.24	95.88	88.82	92.65
P65	98.53	97.06	95.59	98.53	92.65	97.06
P70	100.00	98.53	97.06	100.00	93.09	98.53
P75	100.00	100.00	98.53	100.00	95.59	100.00
P80	100.00	100.00	100.00	100.00	98.53	100.00
P85 ~ P95	100.00	100.00	100.00	100.00	100.00	100.00

### The cut-off value of HHBQ-C for medical staff in tertiary general hospitals in China was established

This research analyzed HHBQ-C score values according to gender and age demarcations, as shown in supplementary material. Norms were established using 10 demarcation schemes, and their relationships to the overall HHBQ-C score were assessed. The results showed that Scheme 4 had the largest *r* value and the highest degree of similarity and consistency, as presented in supplementary material. Therefore, the HHBQ-C scores were divided into four categories: scores within [0, $\bar{x}$-s] were classified as “low”; scores within [$\bar{x}$-s, $\bar{x}$-0.5 s] as “medium”; scores within [$\bar{x}$-0.5 s, $\bar{x}$+0.5 s] as “high”; and scores within [$\bar{x}$+0.5 s, $\bar{x}$+s] also as “very high” Based on these cut-off values, the normative thresholds of HHBQ-C for medical staff in tertiary general hospitals were established by gender and age groups, as shown in Table 5.

**Table 5:** Demarcation for HBBQ-C (points, $\bar{x}$).

Boundary value	Female			Male		
	≤30	31 ~	Female > 40	≤30	31 ~	Male > 35
$\bar{x}$-2.5 s	50.66	45.75	55.21	41.73	46.41	41.04
$\bar{x}$-2 s	58.00	53.87	61.46	50.30	53.77	49.65
$\bar{x}$-1.5 s	65.35	61.99	67.72	58.88	61.14	58.26
$\bar{x}$-s	72.70	70.11	73.97	67.45	68.51	66.87
$\bar{x}$-0.5 s	80.05	78.24	80.22	76.03	75.87	75.48
$\bar{x}$+0.5 s	94.74	94.48	92.72	93.18	90.61	92.70
$\bar{x}$+s	102.09	102.60	98.98	101.75	97.97	101.31
$\bar{x}$+1.5 s	109.44	110.72	105.23	110.33	105.34	109.92
$\bar{x}$+2 s	116.79	118.85	111.48	118.90	112.71	118.53
$\bar{x}$+2.5 s	124.14	126.97	117.73	127.48	120.07	127.14

## Discussion

Whether the norm is scientific and representative mainly depends on the scientific rigor of the measurement tool, the adequacy of the sample size, the appropriateness of the sampling method, and the standardization (18) of the data collection and analysis processes. The HHBQ-C used in this study was developed based on the COM-B model (19), which systematically assesses the facilitators and barriers of HHB across three dimensions: ability, opportunity, and motivation. The reliability and validity (8) of the HHBQ-C have been verified by previous empirical studies conducted in China (Tables 6, 7).

**Table 6:** Demarcation norm scheme for HHBQ-C.

Scheme	Low	Medium	High	Very high	*R*-value
Scheme 1	[0, $\bar{x}$-2.5 s]	[$\bar{x}$-2.5 s, $\bar{x}$-0.5 s]	[$\bar{x}$-0.5 s, $\bar{x}$+0.5 s]	[$\bar{x}$+0.5 s, $\bar{x}$+2.5 s]	0.947**
Scheme 2	[0, $\bar{x}$-2.0 s]	[$\bar{x}$-2.0 s, $\bar{x}$-0.5 s]	[$\bar{x}$-0.5 s, $\bar{x}$+0.5 s]	[$\bar{x}$+0.5 s, $\bar{x}$+2.0 s]	0.948**
Option 3	[0, $\bar{x}$-1.5 s]	[$\bar{x}$-1.5 s, $\bar{x}$-0.5 s]	[$\bar{x}$-0.5 s, $\bar{x}$+0.5 s]	[$\bar{x}$+0.5 s, $\bar{x}$+1.5 s]	0.951**
Scheme 4	[0, $\bar{x}$-s]	[$\bar{x}$-s, $\bar{x}$-0.5 s]	[$\bar{x}$-0.5 s, $\bar{x}$+0.5 s]	[$\bar{x}$+0.5 s, $\bar{x}$+s]	0.955**
Scheme 5	[0, $\bar{x}$-2.5 s]	[$\bar{x}$-2.5 s, $\bar{x}$-s]	[$\bar{x}$-s, $\bar{x}$+s]	[$\bar{x}$+s, $\bar{x}$+2.5 s]	0.456**
Scheme 6	[0, $\bar{x}$-2.0 s]	[$\bar{x}$-2.0 s, $\bar{x}$-s]	[$\bar{x}$-s, $\bar{x}$+s]	[$\bar{x}$+s, $\bar{x}$+2.0 s]	0.456**
Scheme 7	[0, $\bar{x}$-1.5 s]	[$\bar{x}$-1.5 s, $\bar{x}$-s]	[$\bar{x}$-s, $\bar{x}$+s]	[$\bar{x}$+s, $\bar{x}$+1.5 s]	0.456**
Scheme 8	[0, $\bar{x}$-2.5 s]	[$\bar{x}$-2.5 s, $\bar{x}$-1.5 s]	[$\bar{x}$-1.5 s, $\bar{x}$+1.5 s]	[$\bar{x}$+1.5 s, $\bar{x}$+2.5 s]	0.327**
Scheme 9	[0, $\bar{x}$-2.0 s]	[$\bar{x}$-2.0 s, $\bar{x}$-1.5 s]	[$\bar{x}$-1.5 s, $\bar{x}$+1.5 s]	[$\bar{x}$+1.5 s, $\bar{x}$+2.0 s]	0.327**
Scheme 10	[0, $\bar{x}$-2.5 s]	[$\bar{x}$-2.5 s, $\bar{x}$-2.0 s]	[$\bar{x}$-2.0 s, $\bar{x}$+2.0 s]	[$\bar{x}$+2.0 s, $\bar{x}$+2.5 s]	0.259**

**Table 7:** HHBQ-C demarcation of norm (points).

Items	Gender	Age (years)	Low	Medium	High	Very high
Overall score	Female	≤30	[0, 72.70]	[72.70, 80.05]	[80.05, 94.74]	[94.74, 100]
		31 ~	[0, 70.11]	[70.11, 78.24]	[78.24, 94.48]	[94.48, 100]
		Female > 40	[0, 73.97]	[73.97, 80.22]	[80.22, 92.72]	[92.72, 100]
	Male	≤30	[0, 67.45]	[67.45, 76.03]	[76.03, 93.18]	[93.18, 100]
		31 ~	[0, 68.51]	[68.51, 75.87]	[76.87, 90.61]	[90.61, 100]
		Male > 35	[0, 66.87]	[66.87, 75.48]	[75.48, 92.70]	[92.70, 100]
Ability score	Female	≤30	[0, 69.62]	[69.62, 78.64]	[78.64, 96.68]	[96.68, 100]
		31 ~	[0, 69.60]	[69.60, 78.74]	[78.74, 97.01]	[97.01, 100]
		Female > 40	[0, 72.12]	[72.12, 80.31]	[80.31, 96.68]	[96.68, 100]
	Male	≤30	[0, 60.90]	[60.90, 72.32]	[72.32, 95.15]	[95.15, 100]
		31 ~	[0, 66.30]	[66.30, 75.46]	[75.46, 93.78]	[93.78, 100]
		Male > 35	[0, 68.20]	[68.20, 76.75]	[76.75, 93.84]	[93.84, 100]
Chance to score	Female	≤30	[0, 70.31]	[70.31, 78.15]	[78.15, 93.82]	[93.82, 100]
		31 ~	[0, 66.30]	[66.30, 75.11]	[75.11, 92.73]	[92.73, 100]
		Female > 40	[0, 69.13]	[69.13, 76.31]	[76.31, 90.66]	[90.66, 100]
	Male	≤30	[0, 65.99]	[65.99, 74.94]	[74.94, 92.84]	[92.84, 100]
		31 ~	[0, 65.96]	[65.96, 73.60]	[73.60, 88.87]	[88.87, 100]
		Male > 35	[0, 64.39]	[64.39, 73.54]	[73.54, 91.84]	[91.84, 100]
Motivation score	Female	≤30	[0, 74.20]	[74.20, 81.66]	[81.66, 96.58]	[96.58, 100]
		31 ~	[0, 71.51]	[71.51, 79.89]	[79.89, 96.64]	[96.64, 100]
		Female > 40	[0, 76.11]	[76.11, 82.38]	[82.38, 94.91]	[94.91, 100]
	Male	≤30	[0, 69.35]	[69.35, 77.91]	[77.91, 95.02]	[95.02, 100]
		31 ~	[0, 68.52]	[68.52, 76.60]	[76.60, 92.76]	[92.76, 100]
		Male > 35	[0, 67.29]	[67.29, 76.08]	[76.08, 93.65]	[93.65, 100]

In terms of sample selection, this study employed a stratified random sampling method to select 2,973 medical workers from 24 tertiary general hospitals across 12 provinces in China as the normal sample. This approach ensured the regional and institutional representativeness of the sample. Regarding data quality control, the study implemented a double independent check mechanism to eliminate questionnaires containing logical contradictions or patterned responses. This process effectively ensured the authenticity and reliability of the data, so that the established HHBQ-C questionnaire for medical staff in tertiary general hospitals in China demonstrated good overall representativeness.

As the most commonly used type in the scale norm system, the mean norm is characterized by high stability and ease of operation, which makes it highly valuable in the application of the scale (20). The mean norm from large-sample data can serve as a reference for evaluating HHBQ-C scores among medical staff in Chinese tertiary hospitals. In practice, total and dimension scores are standardized and compared with the mean norm to assess score levels. Clinical interpretation should consider the specific medical context and related factors.

This study found that medical staff under 30 had the highest HHBQ-C scores, aligning with Ahmed et al. (21) and suggesting greater compliance with HHB among younger staff. In general, the younger the medical staff, the less work experience they have in the hospital. Hospital management typically identifies medical staff with less experience as key targets for hand hygiene training. As a result, younger staff often receive more frequent training and supervision, enhancing their hand hygiene compliance. This study suggests that hospitals should not only train junior staff but also reinforce hand hygiene education and supervision for senior staff to ensure consistent compliance and reduce HCAIs.

Consistent with the findings of Ahmed et al. (21) and Chang et al. (22), nurses had higher HHBQ-C scores than medical staff in other auxiliary positions. These studies confirm that, due to the higher frequency of contact with patients and longer periods of care in clinical practice, nurses’ hand hygiene compliance directly affects the incidence of HCAIs and the quality of medical safety during hospitalization. Nurses have a more intuitive understanding of the potentially serious consequences of nosocomial infections, and this risk perception may strengthen their hand hygiene compliance.

In addition, differences in hand hygiene compliance among nurses may also be attributed to the stricter hand hygiene training, supervision, and management mechanisms implemented by the hospital’s nursing management department. Nursing management departments typically include hand hygiene compliance in nurses’ performance and quality assessment systems, which may contribute to the higher hand hygiene compliance observed among nurses compared to medical staff in other auxiliary positions.

In terms of the effect of work pressure on the HHBQ-C score, medical staff who perceived “Minor impact” or “Not much” had lower HHBQ-C scores than those who perceived a “A very big impact.” One possible explanation is that medical staff who felt that work pressure had a “A very big impact” on HHB may be more cognitively attentive to hand hygiene. As a result, they are more aware of using hand hygiene to reduce the potential risk of infection in the working environment and therefore pay greater attention to clinical hand hygiene practices. From the perspective of stress-behavior theory, hand hygiene may function as a coping mechanism through which medical personnel alleviate perceived pressures related to hospital-acquired infections and reduce clinical risks. Notably, the stress-behavior relationship does not follow a linear pattern—moderate stress could positively influence hand hygiene compliance.

However, Delva et al. (23) reported that lower levels of work stress were a protective factor for HHB among health care workers in low- and middle-income countries in the Caribbean and Latin America. This finding may be related to differences in values across cultural contexts. Based on the results of this study, it is recommended to develop intervention strategies to improve hand hygiene compliance in medical institutions in China within the framework of stress-behavior theory. It is important to focus on establishing a work stress assessment mechanism for medical staff and to dynamically evaluate their stress levels by optimizing work processes and improving the working environment.

In this study, workload showed a significant impact on the HHBQ-C scores of medical staff. Medical staff who perceived that workload had “No impact” on HHB scored higher than those who perceived that workload had a “A very great impact” on HHB. This may be because the former believe that hand hygiene is a medical norm that must be strictly implemented regardless of workload. This perception may motivate them to maintain high hand hygiene compliance at work. In contrast, the latter may be more likely to neglect hand hygiene when they are busy and believe that excessive work is the main reason for the poor implementation of hand hygiene. This perception may reduce their emphasis on hand hygiene and thus affect their HHBQ-C scores. This finding is similar to the study by Chang et al. (24), who showed that there was no correlation between the performance of hand hygiene and the workload of health care workers at low and moderate workloads. However, previous studies (25) have pointed out that hand hygiene compliance decreases with increasing workload. The results of this study suggest that the relationship between workload and hand hygiene compliance is not necessarily a simple linear relationship. In the future, this relationship can be further explored using machine learning algorithms to develop effective measures to improve the hand hygiene compliance of medical staff.

The percentile norm reflects the relative distribution of individuals’ positions within a standardized sample by describing the percentage of people in the norm group who fall below a certain score. A lower percentile rank indicates a lower relative position of the individual (26). In this study, percentile norms were constructed at 5% intervals to quantify the distribution characteristics of HHBQ-C scores among medical staff. The results showed that higher scores corresponded to higher percentile positions, suggesting that there are more promoting factors and fewer obstacles affecting the HHB of medical staff. Therefore, targeted intervention measures should be developed to address obstacle factors and improve hand hygiene compliance among medical staff.

The mean norm and percentile norm can reflect the relative position of HHBQ-C scores of medical staff within the group, but cannot determine the level of individual scores. Therefore, this study constructed 10 boundary schemes with intervals of 0.5 times the standard deviation and a maximum boundary value of 2.5 times the standard deviation. Through analysis and comparison with the total sample scores, Scheme 4, which showed the strongest correlation, was ultimately selected. The influencing factors of hand hygiene among medical staff were then divided into four grades: low, medium, high, and very high. This classification system provides a specific quantitative basis for researchers to evaluate the level of influencing factors of HHB among medical staff.

## Summary

This study established the mean, classification, percentile, and demarcation norms of the HHBQ-C for medical staff in tertiary general hospitals in China. These norms provide a reference framework for systematically evaluating and identifying the factors that promote or hinder HHB among medical staff and serve as a basis for formulating targeted intervention strategies to improve such behavior. In future studies, hospital administrators, infection control teams, and policymakers could integrate the key determinants of hand hygiene practices identified through this scale into staff training programs, performance evaluations, or targeted interventions, thereby potentially enhancing compliance and ultimately reducing HCAIs. It should be noted that, with the development of medical technology and the overall improvement of the professional quality of medical staff, it is recommended to regularly revise and update these norms to ensure their continued clinical applicability and reference value. In addition, due to limitations in research resources and time, this study included only medical staff from tertiary general hospitals. Tertiary general hospitals differ significantly from primary, secondary, and specialized hospitals in terms of resources, training, and supervision related to hand hygiene practices. In the future, further research can expand the assessment of influencing factors and the establishment of standards for HHB among medical staff in primary, secondary, and specialized hospitals. This study also has certain limitations. During the stratified random sampling process, stratification was based solely on the province where the tertiary hospitals were located, which may have resulted in a sample with limited representativeness. Furthermore, as a cross-sectional investigation, this study cannot establish causal relationships. The HHBQ-C is a self-reported outcome measure for assessing factors influencing hand hygiene behavior, which may be subject to recall bias and social desirability effects, potentially leading to overestimated results. Although an online survey method was adopted to mitigate social desirability bias, future studies should explore more objective data collection approaches to further enhance the reliability and validity of the measurement tool and reduce the impact of systematic errors on research outcomes.

## Data Availability

The original contributions presented in the study are included in the article/supplementary material, further inquiries can be directed to the corresponding author.
