# Supplementary material for: To construct the norm of hand hygiene behavior scale for medical staff in tertiary general hospitals in China
Source: Front Med (Lausanne). 2025 Oct 24;12:1656547. doi: 10.3389/fmed.2025.1656547 (PMC12592085; doi:10.3389/fmed.2025.1656547)
Supplement: Supplementary file 1 [file Table_1.DOCX]

supplementary material 1 Demarcation for HBBQ-C (points, $\overline{\text{x}}$)

| **Boundary value** |  | **female** |  |  | **male** |  |
| --- | --- | --- | --- | --- | --- | --- |
|  | **< = 30** | **31 ~** | **Female >40** | **< = 30** | **31 ~** | **Male >35** |
| $\overline{\text{x}}$-2.5s | 50.66 | 45.75 | 55.21 | 41.73 | 46.41 | 41.04 |
| $\overline{\text{x}}$-2s | 58.00 | 53.87 | 61.46 | 50.30 | 53.77 | 49.65 |
| $\overline{\text{x}}$-1.5s | 65.35 | 61.99 | 67.72 | 58.88 | 61.14 | 58.26 |
| $\overline{\text{x}}$-s | 72.70 | 70.11 | 73.97 | 67.45 | 68.51 | 66.87 |
| $\overline{\text{x}}$-0.5s | 80.05 | 78.24 | 80.22 | 76.03 | 75.87 | 75.48 |
| $\overline{\text{x}}$+0.5s | 94.74 | 94.48 | 92.72 | 93.18 | 90.61 | 92.70 |
| $\overline{\text{x}}$+s | 102.09 | 102.60 | 98.98 | 101.75 | 97.97 | 101.31 |
| $\overline{\text{x}}$+1.5s | 109.44 | 110.72 | 105.23 | 110.33 | 105.34 | 109.92 |
| $\overline{\text{x}}$+2s | 116.79 | 118.85 | 111.48 | 118.90 | 112.71 | 118.53 |
| $\overline{\text{x}}$+2.5s | 124.14 | 126.97 | 117.73 | 127.48 | 120.07 | 127.14 |

| **Scheme** | **Low** | **Medium** | **High** | **Very high** | **R-value** |
| --- | --- | --- | --- | --- | --- |
| Scheme 1 | [0,$\overline{\text{x}}$-2.5s] | ($\overline{\text{x}}$-2.5s,$\overline{\text{x}}$-0.5s] | ($\overline{\text{x}}$-0.5s,$\overline{\text{x}}$+0.5s] | ($\overline{\text{x}}$+0.5s,$\overline{\text{x}}$+2.5s] | .947** |
| Scheme 2 | [0,$\overline{\text{x}}$-2.0s] | ($\overline{\text{x}}$-2.0s,$\overline{\text{x}}$-0.5s] | ($\overline{\text{x}}$-0.5s,$\overline{\text{x}}$+0.5s] | ($\overline{\text{x}}$+0.5s,$\overline{\text{x}}$+2.0s] | .948** |
| Option 3 | [0,$\overline{\text{x}}$-1.5s] | ($\overline{\text{x}}$-1.5s，$\overline{\text{x}}$-0.5s] | ($\overline{\text{x}}$-0.5s,$\overline{\text{x}}$+0.5s] | ($\overline{\text{x}}$+0.5s,$\overline{\text{x}}$+1.5s] | .951** |
| Scheme 4 | [0,$\overline{\text{x}}$-s] | ($\overline{\text{x}}$-s,$\overline{\text{x}}$-0.5s] | ($\overline{\text{x}}$-0.5s,$\overline{\text{x}}$+0.5s] | ($\overline{\text{x}}$+0.5s,$\overline{\text{x}}$+s] | .955** |
| Scheme 5 | [0,$\overline{\text{x}}$-2.5s] | ($\overline{\text{x}}$-2.5s,$\overline{\text{x}}$-s] | ($\overline{\text{x}}$-s,$\overline{\text{x}}$+s] | ($\overline{\text{x}}$+s,$\overline{\text{x}}$+2.5s] | .456** |
| Scheme 6 | [0,$\overline{\text{x}}$-2.0s] | ($\overline{\text{x}}$-2.0s,$\overline{\text{x}}$-s] | ($\overline{\text{x}}$-s,$\overline{\text{x}}$+s] | ($\overline{\text{x}}$+s,$\overline{\text{x}}$+2.0s] | .456** |
| Scheme 7 | [0,$\overline{\text{x}}$-1.5s] | ($\overline{\text{x}}$-1.5s,$\overline{\text{x}}$-s] | ($\overline{\text{x}}$-s,$\overline{\text{x}}$+s] | ($\overline{\text{x}}$+s,$\overline{\text{x}}$+1.5s] | .456** |
| Scheme 8 | [0,$\overline{\text{x}}$-2.5s] | ($\overline{\text{x}}$-2.5s,$\overline{\text{x}}$-1.5s] | ($\overline{\text{x}}$-1.5s,$\overline{\text{x}}$+1.5s] | ($\overline{\text{x}}$+1.5s,$\overline{\text{x}}$+2.5s] | .327** |
| Scheme 9 | [0,$\overline{\text{x}}$-2.0s] | ($\overline{\text{x}}$-2.0s,$\overline{\text{x}}$-1.5s] | ($\overline{\text{x}}$-1.5s,$\overline{\text{x}}$+1.5s] | ($\overline{\text{x}}$+1.5s,$\overline{\text{x}}$+2.0s] | .327** |
| Scheme 10 | [0,$\overline{\text{x}}$-2.5s] | ($\overline{\text{x}}$-2.5s,$\overline{\text{x}}$-2.0s] | ($\overline{\text{x}}$-2.0s,$\overline{\text{x}}$+2.0s] | ($\overline{\text{x}}$+2.0s,$\overline{\text{x}}$+2.5s] | .259** |

supplementary material 2 emarcation norm scheme for HHBQ-C
